# GPCR Signaling Regulation: The Role of GRKs and Arrestins

**DOI:** 10.3389/fphar.2019.00125

**Published:** 2019-02-19

**Authors:** Vsevolod V. Gurevich, Eugenia V. Gurevich

**Affiliations:** Department of Pharmacology, Vanderbilt University, Nashville, TN, United States

**Keywords:** GPCR, GRK, arrestin, signaling, protein engineering

## Abstract

Every animal species expresses hundreds of different G protein-coupled receptors (GPCRs) that respond to a wide variety of external stimuli. GPCRs-driven signaling pathways are involved in pretty much every physiological function and in many pathologies. Therefore, GPCRs are targeted by about a third of clinically used drugs. The signaling of most GPCRs via G proteins is terminated by the phosphorylation of active receptor by specific kinases (GPCR kinases, or GRKs) and subsequent binding of arrestin proteins, that selectively recognize active phosphorylated receptors. In addition, GRKs and arrestins play a role in multiple signaling pathways in the cell, both GPCR-initiated and receptor-independent. Here we focus on the mechanisms of GRK- and arrestin-mediated regulation of GPCR signaling, which includes homologous desensitization and redirection of signaling to additional pathways by bound arrestins.

## GPCR Signaling Via G Proteins

G protein-coupled receptors are the largest family of signaling proteins. Structurally, the cores of all GPCRs are very similar: extracellular N-terminus, seven membrane-spanning α-helices (TM), and intracellular C-terminus, with variable extracellular and intracellular elements (Bockaert and Pin, 1999; Fredriksson et al., 2003). GPCRs mediate cellular response to various stimuli, from light and odorants to hormones, neurotransmitters, and even extracellular protease activity and calcium (Bockaert and Pin, 1999). GPCRs are usually localized on the plasma membrane, serving as “eyes and ears” of the cell. Accordingly, GPCRs have ligand-binding elements exposed on the extracellular side. Most receptors belong to class A that have a ligand-binding pocket between the helices, which could be either close to the extracellular surface or buried almost to half the depth of the membrane (Fredriksson et al., 2003). Even covalently attached 11-cis-retinal in the light receptor rhodopsin sits in a pocket similar to those of the receptors that bind dissociable small molecule ligands. Class B GPCRs interact with peptides. These receptors have large N-termini, which contain the high-affinity part of the ligand-binding site, with the pocket between helices constituting the lower affinity part. Class C GPCRs are dimers, consisting of two 7TM units, with ligand-binding pocket localized on a separate extracellular Venus flytrap domain homologous to bacterial proteins involved in transporting amino acids and ions (Fredriksson et al., 2003; Pin and Bettler, 2016). In class C GPCRs many allosteric modulators bind to the pocket between helices in the 7TM part, so that after the deletion of the extracellular elements the remaining heptahelical domain functions pretty much like a class A receptor binding allosteric regulators as ligands (Goudet et al., 2004). GPCR orthosteric (i.e., binding to the site natural ligand occupies) ligands fall into three categories: activating (agonists), inactivating (inverse agonists that suppress constitutive activity), and neutral (antagonists, that occupy the site but do nothing else). Most natural ligands are agonists, but some are antagonists (e.g., agouti and agouti-related peptide are antagonists of melanocortin receptors). Covalently attached to rhodopsin 11-cis-retinal is an inverse agonist, which ensures virtually zero constitutive activity of rhodopsin in the dark, whereas a photon of light converts it into all-*trans*-retinal, which is an agonist that also remains covalently attached.

G protein-coupled receptor activation is accompanied by the outward movement of transmembrane helices V and VI, which creates a cavity on the cytoplasmic side of the heptahelical domain (Farrens et al., 1996). Activation-induced movement appears to be smaller in case of Gi-coupled GPCRs, as compared to Gs-coupled (Rasmussen et al., 2007, 2011a,b; Koehl et al., 2018; Van Eps et al., 2018). Recent structural studies showed that this cavity serves as a docking site for the heterotrimeric G proteins of all subtypes (Scheerer et al., 2008; Rasmussen et al., 2011b; Szczepek et al., 2014; Carpenter et al., 2016; Liang et al., 2017; Zhang et al., 2017; Koehl et al., 2018; Van Eps et al., 2018). Agonist-activated GPCRs act as guanyl nucleotide exchange factors for heterotrimeric G proteins. In the receptor-bound G protein its nucleotide-binding pocket opens (Oldham and Hamm, 2008; Mahoney and Sunahara, 2016), which results in the loss of GDP occupying this site in the inactive form, and binding of GTP, which is much more abundant in the cytoplasm (Traut, 1994). GTP-liganded G protein α-subunit then dissociates from the receptor and βγ-subunit, whereupon both G protein subunits bind their respective effectors. Freed active receptor can bind and activate another G protein molecule, which provides signal amplification at this level. The effectors of G proteins are either ion channels or enzymes, so that the activation of a single effector molecule induces the movement of numerous ions across plasma membrane or the conversion of many substrate molecules into product, providing further signal amplification. Among GPCR-driven systems signal amplification is the greatest in rod photoreceptors, which gives these cells single photon sensitivity (Baylor et al., 1979). However, everything the cell does costs energy. So, as soon as the cell gets the message, it makes biological sense to stop signaling. In case of GPCRs, rapid signal turnoff is accomplished by a conserved two-step mechanism: receptor phosphorylation by GRKs followed by arrestin binding (Carman and Benovic, 1998).

## Phosphorylation of Active Receptors by GRKs

Activation-induced rhodopsin phosphorylation was discovered in the early 1970s (Kühn and Dreyer, 1972; Kühn, 1974), before it became clear that there is a GPCR family to which rhodopsin belongs. Subsequent studies revealed that rhodopsin kinase binds rhodopsin-containing membranes only upon its activation by illumination (Kuhn, 1978). Later another receptor kinase, originally named β-adrenergic receptor kinase, was discovered, which specifically phosphorylated activated β2-adrenergic receptor (β2AR) (Benovic et al., 1986b). That kinase was also shown to phosphorylate rhodopsin in activation-dependent manner (Benovic et al., 1986a). The cloning and sequencing of this kinase (modern systematic name GRK2, whereas rhodopsin kinase is now called GRK1 Gurevich et al., 2012) suggested that there is a family of GRKs likely targeting different GPCRs (Benovic et al., 1989).

The key question that needed to be answered was why these kinases specifically phosphorylate active receptors, whereas other protein kinases known at the time simply recognized specific sequences within targeted proteins. This question was answered in the visual system, where by proteolysis one could eliminate the rhodopsin C-terminus with all phosphorylation sites, while leaving the rest of the rhodopsin molecule as a functional light receptor. Rhodopsin kinase was shown to be activated by physical interaction with light-activated rhodopsin, whereupon it could phosphorylate anything accessible, including exogenous peptides (Palczewski et al., 1991a). Similar activation mechanism was described for GRK2 (Chen et al., 1993). Apparently, when GRK binds a full-length GPCR, the receptor activates it. Parts of that same receptor happen to be in the vicinity and are therefore phosphorylated by the kinase. However, when the receptor is crowded, like rhodopsin in rod disk membranes, where rhodopsin molecules cover about half of the area, inactive receptors can come close by diffusion and get phosphorylated by the kinase activated by the active receptor (Binder et al., 1990; Binder et al., 1996). This high-gain phosphorylation, which is best observed when a very small fraction of rhodopsin molecules is activated, essentially confirms the mechanism of GRK activation. This mechanism, the phosphorylation of inactive pigment molecules by GRK1 activated by bleached rhodopsin, was further supported by detected phosphorylation of an inactive cone pigment co-expressed in rods with rhodopsin (Shi et al., 2005).

GRKs are soluble proteins, so they need specific mechanisms to bring them to the vicinity of membrane-embedded GPCRs. Visual GRK-1 and -7 are prenylated at their C-termini, which ensures their constitutive membrane localization. The pleckstrin homology (PH) domain of GRK2/3 binds Gβγ (Koch et al., 1993; Touhara et al., 1994). GRK2 was even crystallized in complex with Gβγ bound to its PH domain (Lodowski et al., 2003). The Gβγ binding recruits GRK2/3 to the membrane, where the receptors reside. Thus, Gβγ released after G protein activation by a GPCR helps to recruit GRK2/3 to the receptor to shut off signaling (Haga and Haga, 1992; Pitcher et al., 1992, 1995; Li et al., 2003). It has recently been reported that the dopamine D2 receptor can recruit GRK2 even without G protein activation (Pack et al., 2018), although this observation is puzzling considering that no agonist-dependent phosphorylation of the receptor was observed. Interestingly, the expression of the plekstrin homology domain of GRK2 separately from the rest of the molecule suppresses Gβ-mediated signaling in the cell (Inglese et al., 1994). The GRK4/5/6 subfamily lacks the PH domain as well as the C-terminal prenylation (Gurevich et al., 2012). Instead, the GRKs of this subfamily associate with the plasma membrane via palmitoylation of their C-terminal cysteines and/or via an amphipathic helix interacting with the membrane phospholipids (Gurevich et al., 2012 and references therein).

Interestingly, out of three branches of the GRK family, The GRK1/7, GRK2/3, and GRK/4/5/6, only the first two appear to be activated exclusively by the binding to active GPCRs. GRK4 constitutively phosphorylates the dopamine D1 receptor (Rankin et al., 2006). GRK5 was shown to be activated by phospholipids *in vitro* (Kunapuli et al., 1994) and GRK5 and closely related GRK6 phosphorylate even inactive GPCRs both *in vitro* and in live cells (Tran et al., 2004; Baameur et al., 2010; Li et al., 2015). However, the structural data on the GRK-GPCR complexes suggest that GRK1 (He et al., 2017), as well as GRK5 (He et al., 2017; Komolov et al., 2017) engage the same inter-helical cavity in active GPCRs that is part of the docking site of G proteins and arrestins. In agreement with this, engineered phosphorylation-independent arrestin-2 was shown to compete with GRK2 for the β2AR (Pan et al., 2003), indicating that the binding sites on GPCRs used by GRKs and arrestins bind overlap and include the cavity on the cytoplasmic side of GPCRs that opens upon receptor activation (Farrens et al., 1996).

**FIGURE 1:**
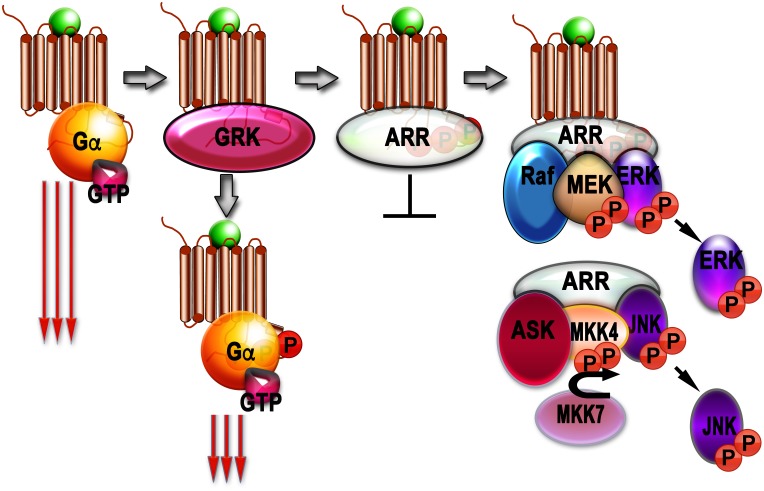
Signaling by G protein-coupled receptors (GPCRs) and arrestins. Agonist-activated GPCRs (agonist is shown as a green ball) bind heterotrimeric G proteins, serving as GEFs: they facilitate the release of GDP bound to the α-subunit of inactive heterotrimer, which subsequently bind GTP. Then Gα subunit dissociates from the GPCR and Gβγ dimer, and both GTP-liganded α-subunit and released Gβγ activate or inhibit various signaling pathways (this signaling is shown as three long arrows). GRKs also bind agonist-activated GPCrs and phosphorylate them. This reduces G protein coupling of active GPCR (three shorter arrows), but complete blockade of G protein-mediated signaling requires arrestin binding to the active phosphorylated GPCR, where arrestins outcompete G proteins. The arrestin-receptor complex acts as a scaffold facilitating different branches of signaling (Raf-MEK-ERK cascade is shown as an example). Free arrestins in the cytoplasm also act as scaffolds, facilitating signaling independently of GPCRs (ASK-MKK4/7-JNK cascade shown as an example).

Although phosphorylation of rhodopsin (Arshavsky et al., 1985) and β2AR (Sibley et al., 1986; Benovic et al., 1989) reduced signaling via G proteins, it did not stop it. So, another set of players was suspected. These players turned out to be arrestins (Figure 1).

## Arrestins Block G Protein Coupling

Preferential binding of arrestins to their cognate receptors when they are active and phosphorylated at the same time was demonstrated directly in case of visual arrestin-1 (Wilden et al., 1986) and non-visual arrestin-2 (Krasel et al., 2005). The role of arrestin-1 (called 48 kDa protein at the time of discovery) in preventing the coupling of phosphorylated rhodopsin to its cognate G protein, transducin, was established in mid-1980s (Wilden et al., 1986). Later is shown independently by two labs that visual arrestin-1 does that by successfully competing with transducin for the light-activated phosphorylated rhodopsin (Wilden, 1995; Krupnick et al., 1997a). The need of an arrestin-like protein in the homologous desensitization of β2AR was shown using purified receptor and GRK2 of different levels of purity. It turned out that while highly purified GRK2 phosphorylated the receptor better than partially purified preparation, it failed to significantly suppress its coupling to the cognate G protein, Gs (Benovic et al., 1987). The addition of purified visual arrestin (arrestin-1 in current systematic nomenclature) significantly enhanced the desensitizing effect of receptor phosphorylation by GRK2, which suggested that non-visual homolog of arrestin-1 might be required for homologous desensitization of the non-rhodopsin GPCRs (Benovic et al., 1987). Soon thereafter the first non-visual arrestin was cloned (Lohse et al., 1990). It was termed β-arrestin because it clearly preferred β2AR over rhodopsin (Lohse et al., 1990, 1992). The second non-visual arrestin was cloned soon after the first, and called β-arrestin2, whereas the first one was retroactively renamed β-arrestin1 (Attramadal et al., 1992). The second non-visual subtype was also cloned from human thyroid and named hTHY-ARRX (Rapoport et al., 1992). When it was cloned for the third time, a systematic arrestin nomenclature, with the number indicating the order of cloning, was proposed, which made this member of the family arrestin-3 (Sterne-Marr et al., 1993). Interestingly, only one additional arrestin, cone photoreceptor-specific arrestin-4, was found in mammals (Murakami et al., 1993; Craft et al., 1994). Thus, hundreds of GPCR subtypes expressed by most mammals (from ∼500 in dolphins and ∼800–1,200 in primates including humans to >3,400 in elephants; sevens.cbrc.jp), are served by only four arrestin proteins, two of which (arrestin-1 and -4) are specialized visual, they are expressed in photoreceptor cells in the retina and bind photopigments, leaving the two non-visual subtypes for the rest of GPCRs. The role of arrestins in terminating G protein-mediated GPCR signaling is well established (Carman and Benovic, 1998). Recent structural data revealed the molecular basis of the competition between G proteins and arrestins: both engage the same inter-helical cavity on the cytoplasmic side of the receptor (Rasmussen et al., 2011b; Kang et al., 2015; Carpenter et al., 2016; Liang et al., 2017; Zhang et al., 2017; Zhou et al., 2017), so that the binding of one precludes the binding of another. Bound G proteins readily dissociate from the receptor in the presence of GTP, whereas arrestins do not. Thus, in case of active phosphorylated GPCRs, which arrestins bind with high affinity (Gurevich and Gurevich, 2004), arrestins easily win in the competition with G proteins. However, in addition to the inter-helical cavity, which is a shared docking site of G protein and arrestins, the latter tightly bind receptor-attached phosphates that fit into positive patches on the arrestin surface (Zhou et al., 2017). This dual-site binding, predicted in 1993 based on arrestin mutagenesis (Gurevich and Benovic, 1993), creates a possibility that arrestin might engage the receptor via only one site. Indeed, it was shown that, at least in case of mutant GPCRs and/or arrestins, the latter can engage solely the phosphorylated receptor C-terminus, leaving the inter-helical cavity accessible for a G protein (Kumari et al., 2016, 2017; Thomsen et al., 2016; Cahill et al., 2017). In this situation “super-complexes” that include a single GPCR simultaneously interacting with G protein and arrestin were observed (Thomsen et al., 2016). Recent study of different subtypes of neuropeptide Y receptors suggests that this mechanism might operate in case of at least some wild type GPCRs (Wanka et al., 2018). However, it appears that simultaneous arrestin interaction with both inter-helical cavity and phosphorylated parts of the receptor, which precludes G protein binding, is the rule, rather than an exception. This mode of arrestin binding is the basis of homologous GPCR desensitization, ensuring direct competition of arrestins with G proteins (Wilden, 1995; Krupnick et al., 1997a).

Both non-visual arrestin subtypes effectively bind clathrin (Goodman et al., 1996) and its adaptor AP2 (Laporte et al., 1999) via specific sites in their C-termini (Kim and Benovic, 2002), which are made more accessible by the release of the C-terminus upon GPCR binding (Zhuo et al., 2014), similar to the rhodopsin binding-induced release of the C-terminus of visual arrestin-1 (Palczewski et al., 1991b; Gurevich et al., 1994; Vishnivetskiy et al., 2002, 2010; Hanson et al., 2006). Thus, non-visual arrestins not only block receptor coupling to the G proteins, but also facilitate GPCR internalization via coated pits (reviewed in Gurevich and Gurevich, 2003), further reducing cell responsiveness. Interestingly, visual arrestin-1 does not have a clathrin-binding site (Goodman et al., 1996), although it has a relatively low affinity AP2 binding site (Moaven et al., 2013). It is likely a relic, as all arrestins apparently arose from an ancestral universal form, similar to a single arrestin in ascidian *Ciona officinalis*, which serves as visual in the eyes of its tadpole and as non-visual in the sessile blind adult (Gurevich and Gurevich, 2006), where it likely promotes GPCR internalization (Nakagawa et al., 2002).

## Arrestin-Mediated Signaling

In addition to stopping (“arresting,” hence the name) GPCR signaling via G proteins, in the last two decades arrestins have been proposed to serve as signal transducers in their own right [reviewed in Gurevich and Gurevich (2006); Hanson et al. (2006); Peterson and Luttrell (2017); Figure 1]. The first signaling function of arrestins was described in 1999: receptor-bound arrestins were found to promote Src-dependent activation of pro-proliferative MAP kinases ERK1/2 (Luttrell et al., 1999). Soon arrestin-3 (but not closely related arrestin-2) was found to scaffold ASK1-MKK4/7-JNK3 cascade, also in receptor-dependent manner (McDonald et al., 2000). Then both non-visual arrestins upon GPCR binding were shown to scaffold yet another three-tiered MAP kinase cascade, c-Raf1-MEK1-ERK1/2 (Luttrell et al., 2001). This finding revealed a previously unappreciated mechanism of GPCR-dependent facilitation of ERK1/2 activation. The number of non-receptor binding partners of non-visual arrestins kept increasing, culminating in a comprehensive proteomics study that described more than a hundred proteins that bind each of the non-visual subtypes, many of which are bona fide signaling proteins (Xiao et al., 2007). The results of protein knockdown using siRNAs even suggested that arrestin-mediated signaling to ERK1/2 is G protein-independent (Shenoy et al., 2006). However, numerous pathways lead to the activation of ERKs (Luttrell, 2003), and many of them, such as receptor tyrosine kinase-dependent (Marshall, 1995), are not even GPCR-driven. Recent findings indicate that in total absence of G protein activity (“zero functional G cells”) due to genetic knockout of members of Gs, Gq, and G12/13 families and inactivation of Gi family members by pertussis toxin, arrestin-mediated signaling in response to GPCR activation cannot be detected (Alvarez-Curto et al., 2016; Grundmann et al., 2018). In contrast, in arrestin-2/3 knockout cells ERK1/2 phosphorylation in response to the activation of several GPCRs, which was often considered a hallmark of arrestin-mediated signaling, is similar to that in parental cells with full complement of non-visual arrestins (O’Hayre et al., 2017). These data are incompatible with the idea that arrestin-mediated signaling is G protein-independent, but do not contradict the notion that arrestin-mediated signaling actually exists, as was shown yet again by a recent study that used three independently generated arrestin-2/3 knockout cell lines (Luttrell et al., 2018). In fact, the role of arrestins in enhancing ERK1/2 activation in the presence of G proteins in case of some GPCRs was documented in the study designed to demonstrate G protein dependence of GPCR signaling (Grundmann et al., 2018).

The major aspect overlooked in virtually all studies of arrestin-mediated signaling is signal initiation (Gurevich and Gurevich, 2018). MAPK kinase activation cascades are highly conserved three-tier signaling modules consisting in general terms of upstream MAP3Ks, intermediate MAP2Ks, and downstream MAPKs. MAP3Ks and MAP2Ks activate their downstream target kinases by phosphorylating their activation loops (Tian and Harding, 2014). MAPKs ultimately phosphorylate various nuclear and cytoplasmic proteins to elicit cellular response. Scaffold proteins, such as arrestins, bring the kinases close to each other, thereby facilitating signal transduction. However, the signaling only occurs when the upstream-most MAP3Ks are activated, and arrestins were never implicated in this event. It is entirely possible that before arrestin-mediated scaffolding has a chance to facilitate signaling, MAP3Ks must be activated by arrestin-independent mechanisms, which can be G protein-dependent in case of GPCRs, or G protein- and GPCR-independent in case of growth factor receptors (Garrington and Johnson, 1999) and integrins (Stupack and Cheresh, 2002). This aspect of arrestin-mediated scaffolding needs to be studied experimentally.

## GPCR-Independent Signaling Functions of GRKs

GRKs have been reported to phosphorylate and thus regulate via phosphorylation numerous non-GPCR substrates, including receptor tyrosine kinases, single transmembrane domain serine/threonine kinases, death receptors, toll-like receptors, transcription factors, various adapter proteins, cytosolic, nuclear and cytoskeletal proteins (for review see Gurevich et al., 2012). Although in most cases the functional role of regulation via GRK-dependent phosphorylation remains poorly understood, the mere number of targets suggests that, in addition to playing the key role in controlling the GPCR signaling, GRKs might play important roles in cell growth, attachment and motility, cell death, proliferation and survival, immunity, cancer, as well as other pathological conditions. One example of such regulation is GRK5-dependent phosphorylation of class II histone deacetylase 5 (HDAC5), which plays a role in pathological cardiac hypertrophy (Martini et al., 2008; Traynham et al., 2016). Another interesting substrate of GRK-dependent phosphorylation is the synuclein family comprising α-, β-, and γ-synucleins, small proteins with poorly defined functions. GRK2 preferentially phosphorylates α- and β-synucleins, whereas α-synuclein is the best substrate of GRK5 (Pronin et al., 2000). The great importance of α-synuclein, which is enriched in the presynaptic terminals in the nervous system, stems from its role in sporadic Parkinson’s disease as the main component of Lewy bodies, a hallmark feature of the disorder, as well as from its genetic association with a familial form of the disease (Polymeropoulos et al., 1997; Benskey et al., 2016).

In addition to regulating multiple non-GPCR signaling pathways via phosphorylation, GRKs can control signaling in phosphorylation-independent manner via direct protein-protein interaction. The best studied mode of such regulation is via the function of the GRK RGS homology (RH) domain. The RH domain of GRKs 2/3 acts in a manner similar to other RGS proteins by binding active Gαq/11 (Siderovski et al., 1996). In contrast to canonical RGS proteins, RH domains of GRKs possess only weak ability to activate intrinsic GTPase of G proteins, but instead reduce the Gq/11-mediated signaling mostly by sequestering active Gαq/11 or directly blocking the receptor (Carman et al., 1999; Dhami et al., 2002; Ribeiro et al., 2009). Although all GRK isoforms are equipped with the RH domain, only the RH domain of GRKs 2/3 appears to be functional, whereas those of other GRKs seem to be unable to interact with any G protein, for they are missing the key binding residues (Carman et al., 1999; Picascia et al., 2004; Sterne-Marr et al., 2004; Lodowski et al., 2006).

GRKs 2/3 possess the PH domain in their C-terminus capable of binding Gβγ (Touhara et al., 1994). In addition to mediating the recruitment of the kinases to the plasma membrane upon receptor activation, it also regulates the Gβγ-dependent signaling in the same manner as RH domain regulates the Gq/11-mediated signaling: by sequestering Gβγ and inhibiting Gβγ-mediated signaling processes. This mechanism has been described for G protein-coupled inwardly rectifying potassium channels (GIRK) activated by adenosine A1 and μ-opioid receptors (Raveh et al., 2010) and for κ-opioid receptors (Abraham et al., 2018). GRKs are able to perform other protein binding and/or scaffolding functions (for details see Gurevich et al., 2012). For example, GRK5 biding to calmodulin assists in the nuclear translocation associated with the cardiac hypertrophy (Gold et al., 2013). Furthermore, in addition to phosphorylating HDAC5 in the nucleus, GRK5 contributes to pathological cardiac hypertrophy by activating nuclear factor of activated T cells (NFAT) transcription factor in phosphorylation-independent manner via direct binding (Hullmann et al., 2014). Thus, GRK functions requiring the kinase activity and phosphorylation-independent actions go hand in hand in physiological and pathological processes.

Some of these functions are mediated by GRK interacting proteins (GITs), which are themselves large multidomain scaffolding proteins interacting with multiple partners and playing important role in numerous cellular processes (Premont et al., 1998; Hoefen and Berk, 2006). The involvement of GRKs via their phosphorylation-independent scaffolding function in multiple signaling events in cells led to a suggestion that GRKs might, in concert with arrestins, serve as a critical node within the complex signaling network and a play a role in multiple conditions and/or pathologies, such as aging, cardiovascular and neurodegenerative disorders (Hendrickx et al., 2018).

## GPCR-Independent Signaling Functions of Arrestins

Arrestins also serve as signaling molecules independently of GPCRs (Figure 1). Arrestin-3-dependent facilitation of JNK3 activation was first shown to be independent of GPCRs as early as 2001 (Miller et al., 2001). This was later confirmed by the use of arrestin mutants that do not bind receptors (Song et al., 2009a; Breitman et al., 2012) and by reconstitution of MKK4/7-JNK3 modules with arrestin-3 using purified proteins *in vitro* in the absence of GPCRs (Zhan et al., 2011; Kook et al., 2014b). A short arrestin-3-derived peptide lacking most known receptor-binding elements was found to facilitate JNK3 activation *in vitro* and in living cells (Zhan et al., 2016). Interestingly, the measurements of the affinities of active and inactive kinases of the ASK1-MKK4/7-JNK3 cascade suggest that the activation of MKK4, MKK7, and JNK3 by phosphorylation reduces their binding to arrestin-3 (Perry et al., 2019). This reduction in binding affinity is the most striking in case of JNK3, suggesting that activated (doubly phosphorylated) JNK3 likely dissociates, freeing the place for another molecule of inactive JNK3 to bind (Perry et al., 2019). This “conveyor belt” mechanism allows the complex of arrestin-3 with upstream kinases to sequentially activate several JNK3 molecules, thereby amplifying the signal (Perry et al., 2019). It would be interesting to test whether other MAP kinase scaffolds also employ similar amplification mechanism. The critical role of caspase-cleaved arrestin-2 in programmed cell death also did not appear to depend on receptor binding (Kook et al., 2014a). Caspase cleavage of the other non-visual subtype, arrestin-3, which generates anti-apoptotic arrestin-3-(1-366) fragment (Kook et al., 2019), also does not require GPCR activation. The function of both non-visual arrestins in focal adhesion disassembly (Cleghorn et al., 2015) or in the activation of small G proteins that regulate cytoskeleton (Cleghorn et al., 2018) also did not require arrestin interactions with the receptor.

Thus, widespread belief that arrestin-mediated signaling is always GPCR-driven appears to be wrong. In fact, there are known non-receptor partners that free arrestins, including arrestin mutants that do not interact with GPCRs, bind with higher affinity than receptor-bound (presumably “active”) arrestins. These include E3 ubiquitin ligases Mdm2 (Song et al., 2006) and parkin (Ahmed et al., 2011), whereas all functional forms of arrestin-3 appear to bind JNK3 comparably (Song et al., 2006). So, the question what is the “active” conformation of arrestins and whether there are several “active” conformations facilitating different branches of signaling, needs to be answered experimentally.

## Potential Routes of Intervention

Elucidation of every molecular mechanism of cellular functions paves the way to devising tools that affect this mechanism for therapeutic purposes. GPCR desensitization via GRKs and arrestins, as well as arrestin-dependent signaling are no exceptions. Arguably, congestive heart failure is the most studied condition where GPCR desensitization plays a role in the pathology (reviewed in Lymperopoulos et al., 2012). Heart failure manifests itself as the loss of heart responsiveness to pro-contractile stimuli. This is primarily adrenalin, which acts via β-adrenergic receptors, both β1 and β2 subtypes. Both appear to undergo excessive desensitization mediated by GRK2. When GRK2 ability to suppress β-adrenergic signaling is reduced by overexpression of GRK2 C-terminus that outcompetes endogenous full-length kinase for the G protein βγ-subunit, which targets GRK2 to the plasma membrane where β-adrenergic receptors reside, heart failure is alleviated (Tevaearai et al., 2001). This does not appear to cause many side effects, in contrast to inhibitors of ubiquitously expressed GRK2 (even if selective ones were available), that would likely indiscriminately affect the desensitization of many GPCRs in various cell types. An alternative approach that has not been tested experimentally so far would be to express in the heart one of existing arrestin mutants that binds unphosphorylated β-adrenergic receptors (Gurevich et al., 1997; Kovoor et al., 1999; Celver et al., 2002). At least one of these mutants was shown to directly compete with GRK2 for the receptor and greatly facilitate receptor recycling back to the plasma membrane (Pan et al., 2003), where it can signal.

These “enhanced” phosphorylation-independent versions of non-visual arrestins were also proposed as tools to compensate for excessive GPCR signaling in diseases associated with activating receptor mutations and/or defects in receptor phosphorylation (Stoy and Gurevich, 2015). This approach was so far tested only in the visual system, where enhanced arrestin-1 improved photoreceptor performance and survival in mouse models with defective rhodopsin phosphorylation (Song et al., 2009b; Samaranayake et al., 2018). However, for this purpose enhanced mutants of non-visual arrestins must be rendered specific for particular GPCRs to avoid their effect on the signaling of perfectly normal GPCRs co-expressed in the same cell (Gurevich and Gurevich, 2017). The first attempts to develop receptor-specific non-visual arrestins appear promising, because as few as two mutations on the receptor-binding surface yielded mutants with ∼50–60-fold preference for some GPCRs over others (Gimenez et al., 2012b). Manipulation of the receptor-binding surface of arrestins was also shown to change their selectivity for particular functional forms of the receptor (Prokop et al., 2017). However, the ability of these receptor subtype-specific forms to selectively suppress signaling by certain GPCRs without affecting others co-expressed in the same cell still remains to be tested.

Another therapeutic approach that needs to be tested is the use of signaling-biased arrestins. WT non-visual arrestins have a lot of functions: they bind hundreds of different GPCRs and dozens of non-receptor signaling proteins, affecting multiple branches of cellular signaling (Hanson et al., 2006; Peterson and Luttrell, 2017). Thus, an increase or decrease of WT arrestin expression in cells cannot serve a specific function: too many things would change. However, quite a few arrestin mutants where individual functions are suppressed or destroyed were constructed: forms that do not bind GPCRs [KNC mutants (Breitman et al., 2012; Gimenez et al., 2012a, 2014)], mutants with disabled clathrin and/or AP2 binding sites (Kim and Benovic, 2002), arrestin-3 variant that does not facilitate JNK activation (Seo et al., 2011), arrestin-2 mutants that do not promote ERK1/2 activation due to reduced binding of upstream kinases MEK1 (Meng et al., 2009) or c-Raf1 (Coffa et al., 2011). One drawback is that in some cases mutations affect not only the intended function, but others, as well. For example, arrestin-3-KNC, despite normal or even enhanced binding to the kinases of ASK1-MKK4/7-JNK3 cascade, fails to facilitate the activation of JNK3, and even acts as a silent scaffold, suppressing JNK3 activation by WT arrestin-3 (Breitman et al., 2012). It is hardly practical to test every mutant for all the functions that corresponding WT arrestin fulfills, so these mutants might have limited therapeutic usability. In contrast, monofunctional elements extracted from multi-functional arrestin proteins hold greater promise. So far two of these were constructed. Separated arrestin-2 C-terminus carrying both clathrin and AP2 binding sites, effectively outcompetes the arrestin-receptor complexes in coated pits, suppressing arrestin-dependent GPCR endocytosis in cells (Krupnick et al., 1997b). Arrestin-3 N-terminal peptide (T1A, which is only 25 residues long and lacks most receptor-binding elements) serves as a scaffold for the ASK1/MKK4/7-JNK3 cascade, facilitating JNK3 activation *in vitro* and in cells (Zhan et al., 2016). Thus, at least two arrestin functions can be manipulated independently of the others. Of course, this is just the beginning, but these findings are encouraging.

All of the examples above imply gene therapy, which is currently in its infancy. Theoretically, identification of arrestin sites responsible for the binding of any particular partner enable the search for small molecules that can bind to the site and selectively inhibit an individual interaction. This avenue also needs to be explored, although it might yield fewer useful molecules than one would expect. Protein-protein interaction sites often involve relatively flat surfaces (Gurevich and Gurevich, 2014) or disordered protein elements (Gurevich et al., 2018), both of which are notoriously hard to target with small molecules.

To summarize, selective regulation of GRKs and arrestins appears promising as a therapeutic approach. Many avenues of this regulation, involving both conventional small molecule therapeutics and gene therapy, must be explored.

## Author’s Note

We use systematic names of arrestin proteins, where the number after the dash indicates the order of cloning: arrestin-1 (historic names S-antigen, 48 kDa protein, visual or rod arrestin), arrestin-2 (β-arrestin or β-arrestin1), arrestin-3 (β-arrestin2 or hTHY-ARRX), and arrestin-4 (cone or X-arrestin).

## Author Contributions

VG and EG wrote the manuscript.

## Conflict of Interest Statement

The authors declare that the research was conducted in the absence of any commercial or financial relationships that could be construed as a potential conflict of interest.
